# Sophocarpine Protects Mice from ConA-Induced Hepatitis via Inhibition of the IFN-Gamma/STAT1 Pathway

**DOI:** 10.3389/fphar.2017.00140

**Published:** 2017-03-21

**Authors:** Xiu-Xiu Sang, Rui-Lin Wang, Cong-En Zhang, Shi-Jing Liu, Hong-Hui Shen, Yu-Ming Guo, Ya-Ming Zhang, Ming Niu, Jia-Bo Wang, Zhao-Fang Bai, Xiao-He Xiao

**Affiliations:** ^1^China Military Institute of Chinese Medicine, 302 Military HospitalBeijing, China; ^2^Integrative Medical Center, 302 Military HospitalBeijing, China

**Keywords:** Sophocarpine, Concanavalin A-induced hepatitis, interferon-γ, signal transducers and activators of transcription1, suppressor of cytokine signaling1

## Abstract

Sophocarpine is the major pharmacologically active compound of the traditional Chinese herbal medicine *Radix Sophorae Subprostratae* which has been used in treating hepatitis for years in China. It has been demonstrated that Sophocarpine exerts an activity in immune modulation and significantly decreases the production of inflammatory cytokines. However, the protective effects of Sophocarpine in T cell-dependent immune hepatitis remained unknown. The aim of this study was to determine the protective effects and pharmacological mechanisms of Sophocarpine on Concanavalin A (ConA)-induced hepatitis, an experimental model of T cell-mediated liver injury. BALB/C mice were pretreated with Sophocarpine or Bicyclol for five consecutive days. Thirty minutes after the final administration, the mice were injected with 15 mg⋅kg^-1^ of ConA intravenously. The results indicated that pretreatment with Sophocarpine significantly ameliorated liver inflammation and injury as evidenced by both biochemical and histopathological observations. Moreover, in Sophocarpine-pretreated mice, liver messenger RNA expression levels of chemokines and adhesion molecules, such as macrophage inflammatory protein-1α, CXC chemokine ligand 10, and Intercellular adhesion molecule-1, were markedly reduced. Further studies revealed that Sophocarpine significantly downregulated the expression of T-bet via inhibition of signal transducers and activators of transcription1 (STAT1) activation and overexpression of suppressor of cytokine signaling1, inhibiting the activation of Th1 cells and the expression of Interferon-γ (IFN-γ). Altogether, these results suggest new opportunities to use Sophocarpine in the treatment of T cell-mediated liver disease. In summary, Sophocarpine could attenuate ConA-induced liver injury, and the protective effect of Sophocarpine was associated with its inhibition effect of pro-inflammatory cytokines, chemokines, and the IFN-*γ*/STAT1 signaling pathway.

## Introduction

Hepatitis, which is caused by alcohol drinking, certain drugs, viral infection, or autoimmunity, is a serious threat to human health (Zheng et al., 2016). ConA-induced autoimmune hepatitis has been widely used in studies of immune hepatitis treatment in humans which has been considered a well-established experimental model for immune-mediated liver injury. CD4 T cells were essential to mediate adaptive immunity in response to a variety of pathogens. Intravenous application of the ConA in mice results in the activation of naive CD4 T cells and then subsequent differentiation into T helper cells, including Th1 (secrete IFN-γ), Th2 [secrete Interleukin-4 (IL-4)], Th17 cells (secrete IL-17), and Treg cells (Schumann et al., 2003; Siebler et al., 2003; Stummvoll et al., 2008; Manns et al., 2015). In the pathogenesis of ConA injection, IFN-γ was reported to be critically involved in liver injury because in the mice deficient in IFN-γ or treated with IFN-γ–neutralizing monoclonal antibody its disease progress was significantly reduced (Miyagi et al., 2004). IFN-γ was also reported to activate STAT1, inducing T-bet expression, which is essential for Th1 cell differentiation (Shinohara et al., 2005; Zhu et al., 2010). SOCS1 is a negative regulator of STAT1 activation. It has been reported that overexpression of SOCS1 prevents ConA-induced liver injury by suppressing STAT1 activation (Ogata et al., 2006).

Sophocarpine exhibits many pharmacological activities and is a major matrine-type alkaloid present in the traditional Chinese herb, *Radix Sophorae Subprostratae*. Sophocarpine has been demonstrated to alleviate pain effectively in thermally and chemically induced mouse models (Gao et al., 2009), and it also inhibits LPS-mediated NF-κB activation in RAW 264.7 cells (Gao et al., 2012). Moreover, Sophocarpine can decrease serum levels of aminotransferase and TBIL and alleviate liver fibrosis by inhibiting the TLR4 pathway (Qian et al., 2014). It also exerts an activity in immune modulation (Zhang et al., 2007). Despite the evidence presented by these experimental studies, little is known regarding the effect of Sophocarpine on ConA-induced immunity hepatitis.

The aim of this study was to determine whether Sophocarpine could inhibit immune-mediated liver disease in T cell-dependent hepatitis mouse model. In addition, we aimed to define potential mechanisms by which Sophocarpine exerts its hepatoprotective role in ConA-induced liver injury.

## Materials and Methods

### Mice

Male BALB/C mice, weighing approximately 18 ± 22 g, were acquired from SPF Animals Biotechnology Co., Ltd. (Beijing, China) (Certification number SCXK-JING 2011-0004). The mice were housed in the Institutional Laboratory Animal Care center at 302 Military Hospital. All of the animals were maintained according to the National Institutes of Health Guidelines for Animal Care and the Guidelines of Scripps. The protocol was approved by the Committee on the Ethics of Animal Experiments of the 302 Military Hospital (approval ID: IACUC-2016-008). Efforts were made to minimize animal suffering and to reduce the number of animals used in the experiments. The animals were randomized into Control, Model, Bicyclol (100 mg⋅kg^-1^), Sophocarpine (30 and 60 mg⋅kg^-1^) groups. The drugs were dissolved in saline and orally administered for five consecutive days. All of the mice in the control group were intragastrically administered with an equivalent volume of free saline. Thirty minutes after the final administration of medication, the mice were injected with 15 mg⋅kg^-1^ of ConA intravenously by tail except for the normal group. The mice were sacrificed 8 h after injection. Blood samples were obtained, and the liver was collected simultaneously.

### Blood Chemistry Measurement

The serum levels of ALT, AST, and TBIL were determined using the detection kits provided by Nanjing Jiancheng Bioengineering Institute (Nanjing, China). All procedures were performed according to the manufacturer’s instructions. Serum was diluted fivefold for the detection of aminotransferase.

### Liver Histology, TUNEL Staining, and Immunohistochemistry

Livers were fixed with 4% paraformaldehyde and embedded in paraffin. Next, they were cut into 5 μm-thick sections and the sections were stained with hematoxylin and eosin (H&E) or a TUNEL kit was used (Promega, USA). Immunohistochemistry was performed by liver sections and stained with ICAM-1 antibody. The levels of tissue damage and immune complexes were examined using light microscopy.

### Analysis of Plasma Cytokines

Plasma concentrations of TNF-α, IFN-γ, IL-10, and TGF-β1 were determined using ELISA kits (MultiSciences Biotech Co., Ltd., Hangzhou, China) according to the manufacturer’s instructions. All indexes were measured 8 h after ConA injection.

### Quantitative PCR

Total RNA was isolated from liver tissues by Trizol (Life Sciences) and first-strand cDNA synthesis was performed in 20 μl of solution using the Revert kit (Thermo-Fisher). The mRNA expression level was quantified using real-time PCR (Applied Biosystems 7500 Real-Time PCR System) and a SYBR Green PCR Mix kit (Life Sciences). The primers sequences were as follows: GAPDH, 5′-GTCCTCAGTGTAGCCCAAGAT-3′ and 5′-CAATGTGTCCGTCGTGGATCT-3′; T-bet, 5′-TGCCCGAACTACAGTCACGAAC-3′ and 5′-AGTGACCTCGCCTGGTGAAATG-3′; GATA3, 5′-GGAGGACTTCCCCAAGAGCA-3′ and 5′-CATGCTGGAAGGGTGGTGA-3′; SOCS1, 5′-TCCGATTACCGGCGCATCACG-3′ and 5′-CTCCAGCAGCTCGAAAAGGCA-3′; MIP-1α, 5′-CACCCTCTGTCACCTGCTCAA-3′ and 5′-ATGGCGCTGAGAAGACTTGGT-3′; ICAM-1, 5′-CCATCACCGTGTATTCGTTTCC-3′ and 5′-CTGGCGGCTCAGTATCTCCTC-3′; CXCL10, 5′-TCCAGTTAAGGAGCCCTTTTAGACC-3′ and 5′-TGAAATCATCCCTGCGAGCCTAT-3′. The CT values were then converted to fold induction over normal at time zero (control) using the 2^-ΔΔCT^ formula.

### Isolation of Lymphocytes and Flow Cytometry

1^∗^10^6^ splenocytes from the different groups of experimental mice were stimulated for 6 h with PMA (100 ng/μl), Ionomycin (1 μg/μl), and Monensin (1 μg/μl) and stained with the Percy/cy5.5-conjugated anti-CD3 (Dakewe Biotech Co., Ltd.) and PE/cy-7-conjugated anti-CD8 (Dakewe Biotech Co., Ltd.); fluorescence antibodies specific for surface antigens were used according to the manufacturer’s instructions. For intracellular cytokine staining, the cells were fixed, permeabilized, and stained with PE-conjugated anti-IL-4 (BD Biotech Co., Ltd.) and FITC-conjugated anti-IFN-γ (BD Biotech Co., Ltd.). The stained cells were analyzed using a flow cytometer (BD Canto II), and the data were analyzed using FlowJo 7.6.1.

### Western Blotting Analyses

Liver tissue was lysed on ice with RIPA buffer (G2002) containing a protease inhibitor mixture. After centrifugation, the samples were incubated for 10 min at 4°C to separate any debris. The membrane was incubated with antibodies against β-actin, T-bet, STAT1, and phospho-STAT1 (Tyr705) in TBS/T containing 5% non-fat milk overnight at 4°C. After incubation with the appropriate peroxidase-conjugated secondary antibody, the membrane was washed in TBST for 5 min.

### Statistical Analysis

All results are expressed as the mean ± SD and analyzed using SPSS software (version 20.0; SPSS, Inc., Chicago, IL, USA). Differences were considered to be significant when *p* < 0.05 and highly significant when *p* < 0.01.

## Results

### Effects of Sophocarpine on Serum in ConA-Induced Acute Hepatitis

**FIGURE 1:**
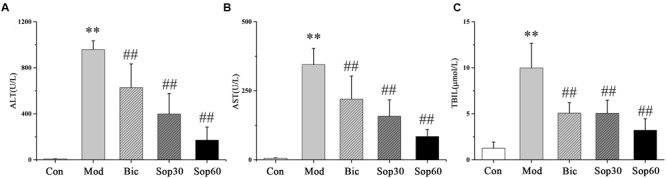
**Effects of Sophocarpine on serum in ConA-induced acute hepatitis.** Female BALB/c mice were pretreated with Sophocaripine (30, 60 mg⋅kg^-1^) and Bicyclol at 30 min before ConA injection. Serum transaminase ALT **(A)** and AST **(B)** TBIL **(C)** levels were determined 8 h after ConA injection. Data is expressed as mean ± SD (*n* = 12; ^∗^*p* < 0.05, ^∗∗^*p* < 0.01 compared with the control group, ^#^*p* < 0.05,^##^*p* < 0.01 compared with the model group, respectively). Saline group (Con group), ConA group (Mod group), Bicyclol group (Bic group), 30 mg⋅kg^-1^of Sophocarpine group (Sop30 group), and 60 mg⋅kg^-1^ of Sophocarpine group (Sop60 group).

To examine the effect of Sophocarpine on ConA-induced acute hepatitis, mice were pretreated with Sophocarpine (30 or 60 mg⋅kg^-1^) for five consecutive days. Bicyclol, as a novel derivative of Bifendate (Yuan et al., 2016) which has been confirmed to decrease the levels of serum ALT served as a positive parallel control, and control mice received saline accordingly. Thirty minutes after the final dose of medication, all the mice were injected with 15 mg⋅kg^-1^ of body weight of ConA via the tail vein except the normal group. Eight hours later, plasma was obtained and serum levels of ALT, AST, and TBIL were measured. Compared with the normal group, the ALT (Control: 8.20 ± 1.24, Model: 957.92 ± 76.46), AST (Control: 5.64 ± 1.45, Model: 344.98 ± 58.50), and TBIL (Control: 1.26 ± 0.66, Model: 9.99 ± 2.67) in the ConA group were significantly increased. Administration of Bicyclol and Sophocarpine inhibited the elevation of ALT [Bicyclol: 628.12 ± 205.94, Sophocarpine (30 mg⋅kg^-1^): 398.02 ± 178.35, Sophocarpine (60 mg⋅kg^-1^): 171.25 ± 113.68], AST [Bicyclol: 219.81 ± 83.58, Sophocarpine (30 mg⋅kg^-1^): 157.69 ± 59.55, Sophocarpine (60 mg⋅kg^-1^): 84.46 ± 25.28], and TBIL [Bicyclol: 5.08 ± 1.13, Sophocarpine (30 mg⋅kg^-1^): 5.05 ± 1.42, Sophocarpine (60 mg⋅kg^-1^): 3.21 ± 1.25] compared with the ConA group (**Figures 1A–C**). Moreover, Sophocarpine inhibited ConA-induced aminotransferase release and TBIL increase in a dose-dependent manner. Taken together, these data significantly show the protective effect of Sophocarpine in ConA-induced hepatitis.

### Sophocarpine Attenuated Immunological Liver Injury in Mice

**FIGURE 2:**
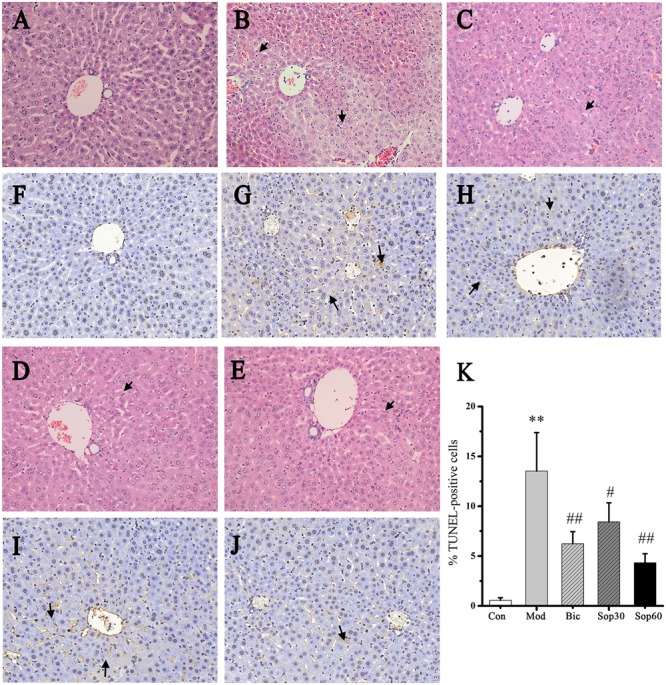
**Sophocarpine attenuated immunological liver injury in mice.** Mice were treated with different doses of Sophocarpine. Representative photographs of six animals show the effect of Sophocarpine, which was confirmed by hematoxylin and eosin (H&E) and TUNEL Staining. HE staining for **(A)** control mice, **(B)** model mice, **(C)** Bicyclol group, **(D)** 30 mg⋅kg^-1^of Sophocarpine group, **(E)** 60 mg⋅kg^-1^of Sophocarpine group. TUNEL staining for **(F)** control mice, **(G)** model mice, **(H)** Bicyclol group, **(I)** 30 mg⋅kg^-1^of Sophocarpine group, **(J)** 60 mg⋅kg^-1^of Sophocarpine group, and **(K)** Percentage of TUNEL-positive cells (^∗∗^*p* < 0.01 compared with the control group, ^#^*p* < 0.05, ^##^*p* < 0.01 compared with the model group, respectively) (H&E, magnification ×200; TUNEL, magnification ×200).

Histopathological examinations of livers showed that, compared with the normal group, the model group revealed severe injury characterized by extensive inflammatory infiltration around the central veins (**Figures 2A,B**). In contrast, mice pretreated with Bicyclol and Sophocarpine demonstrated much less inflammatory infiltration (**Figures 2C–E**). In the Sophocarpine-pretreated group, only a few hepatocytes exhibited TUNEL-positive nuclei compared with the model group (**Figures 2F–K**) [Control: 0.57 ± 0.26, Model: 13.53 ± 3.86, Bicyclol: 6.23 ± 1.22, Sophocarpine (30 mg⋅kg^-1^): 8.43 ± 1.91, Sophocarpine (60 mg⋅kg^-1^): 4.33 ± 0.90], indicating markedly reduced apoptosis in Sophocarpine-pretreated mice. Altogether, these results showed that Sophocarpine dose-dependently inhibited ConA-induced liver inflammation and cell death.

### Sophocarpine Inhibited the Expression of Chemokines and Adhesion Molecules in Mice with ConA-Induced Hepatitis

**FIGURE 3:**
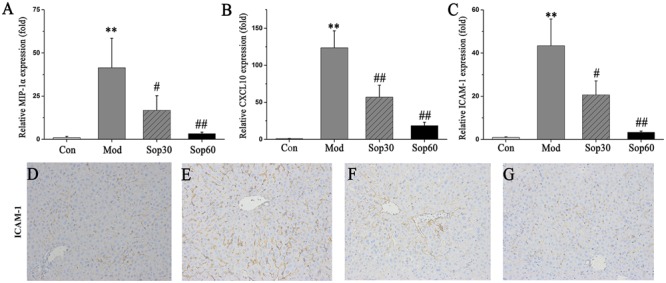
**Sophocarpine inhibited chemokine and adhesion molecule expression in the liver.** BALB/C mice received Sophocarpine pretreatment and ConA injection, 8 h later liver tissues were obtained for quantitatively PCR and immunohistochemistry for ICAM-1. The mRNA expression levels of MIP-1α **(A)**, CXCL10 **(B)**, and ICAM-1 **(C)**. The immunohistochemistry of ICAM-1 for control mice **(D)**, model mice **(E)**, 30 mg⋅kg^-1^of Sophocarpine group **(F)**, 60 mg⋅kg^-1^of Sophocarpine group **(G)** (*n* = 6 each group). ^∗∗^*p* < 0.01 compared with the control group, ^#^*p* < 0.05, ^##^*p* < 0.01 compared with the model group.

In ConA-induced hepatitis, the expression of chemokines and adhesion molecules were demonstrated to play important roles in recruiting lymphocytes into the liver to aggravate liver injury, such as MIP-1α, CXCL10, and ICAM-1 (Ajuebor et al., 2004; Kawasuji et al., 2006; Sahin et al., 2013). Thus, further studies were performed to examine the changes induced by these factors in the mice liver. Liver tissues were obtained 8 h after ConA administration, and mRNA expression levels of MIP-1α, CXCL10, and ICAM-1 were measured. As shown in **Figure 3**, the mRNA levels of MIP-1α [Control: 1.00 ± 0.72, Model: 41.47 ± 17.03, Sophocarpine (30 mg⋅kg^-1^): 16.80 ± 8.45, Sophocarpine (60 mg⋅kg^-1^): 3.32 ± 0.84], CXCL10 [Control: 1.00 ± 0.29, Model: 123.72 ± 22.81, Sophocarpine (30 mg⋅kg^-1^): 57.05 ± 16.03, Sophocarpine (60 mg⋅kg^-1^): 18.56 ± 4.59], and ICAM-1[Control: 1.00 ± 0.19, Model: 43.44 ± 12.33, Sophocarpine (30 mg⋅kg^-1^): 20.69 ± 6.41, Sophocarpine (60 mg⋅kg^-1^): 3.35 ± 0.50] were dramatically reduced by pretreatment with Sophocarpine. Taken together, these results indicate that pretreatment with Sophocarpine significantly reduced ConA-induced intrahepatic inflammatory cell infiltration.

### Sophocarpine Inhibited the Production of IFN-*γ* in Mice with ConA-Induced Hepatitis

**FIGURE 4:**
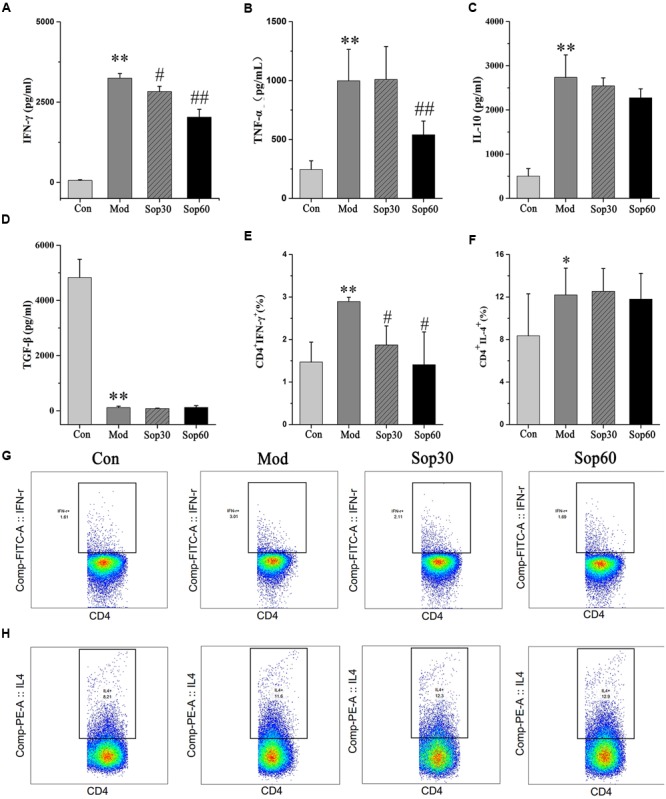
**Sophocarpine inhibited production of IFN-γ in ConA-induced hepatitis mice.** Blood samples were collected at 8 h after ConA injection. Serum levels of cytokines were detected by ELISA. Influences of Sophocaripine on inflammatory cells were measured by Flow Cytometric. Data is expressed as mean ± SD. Serum ELISA for **(A)** IFN-γ, **(B)** TNF-α, **(C)** IL-10, **(D)** TGF-β1. Flow cytometry for **(E)** CD4^+^ IFN-γ^+^, and **(F)** CD4^+^IL-4^+^. Representative photos of each group for **(G)** CD4^+^ IFN-γ^+^, **(H)** CD4^+^IL-4^+^. (*n* = 6; ^∗^*p* < 0.05, ^∗∗^*p* < 0.01 compared with the control group, ^#^*p* < 0.05, ^##^*p* < 0.01 compared with the model group).

It was previously reported that following ConA administration, immune cells were activated. Consequently, various cytokines that aggravated the liver injury were released (Miyagi et al., 2004; Wang et al., 2012). To examine the protective mechanisms of Sophocarpine against liver injury, the effects of Sophocarpine on the serum levels of IFN-γ, TNF-α, IL-10, and TGF-β1 were measured using ELISA. The percentage of CD4^+^IFN-γ^+^ and CD4^+^IL-4^+^ in Sophocarpine-pretreated mice splenocytes were examined using flow cytometry. These results showed that Sophocarpine inhibited the production of IFN-γ [Control: 59.52 ± 23.83, Model: 3248.00 ± 141.02, Sophocarpine (30 mg⋅kg^-1^): 2831.00 ± 160.28, Sophocarpine (60 mg⋅kg^-1^): 2030.00 ± 242.52] and TNF-α [Control: 245.52 ± 73.64, Model: 998.35 ± 266.91, Sophocarpine (30 mg⋅kg^-1^): 1009.78 ± 279.96, Sophocarpine (60 mg⋅kg^-1^): 540.99 ± 116.05] in serum and decreased the percentage of CD4^+^ IFN-γ^+^ [Control: 1.47 ± 0.47, Model: 2.89 ± 0.10, Sophocarpine (30 mg⋅kg^-1^): 1.87 ± 0.45, Sophocarpine (60 mg⋅kg^-1^): 1.41 ± 0.77] in splenocytes compared with the model group (**Figure 4**), but there was no significant difference among the treatment groups in regards to the percentage of CD4^+^IL-4^+^ and the expression of IL-10 and TGF-β1. Therefore, Sophocarpine effectively suppressed the production of IFN-γ and TNF-α and reduced the liver damage caused by these inflammatory cytokines.

### Sophocarpine Downregulated the Expression of T-bet via Inhibition of STAT1 Activation and Overexpression SOCS1

**FIGURE 5:**
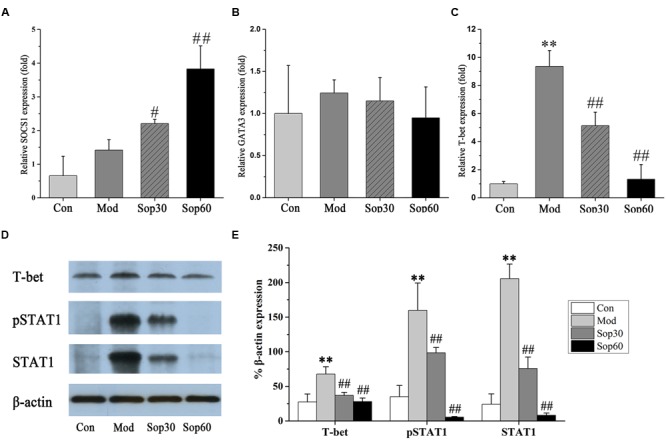
**Sophocaripine downregulated the expression of T-bet via inhibiting STAT1 activation and overexpression of SOCS1.** Mice received Sophocaripine pretreatment and ConA injection as described. The mRNA expression levels of **(A)** SOCS1, **(B)** GATA3, **(C)** T-bet were measured by q-pcr (*n* = 6 each group). The protein expression of **(D)** T-bet, pSTAT1, STAT1 were measured by Western blot. **(E)** The data analysis of Western blot (*n* = 3). ^∗∗^*p* < 0.01 compared with the control group, ^#^*p* < 0.05, ^##^*p* < 0.01 compared with the model group.

It has been previously shown that ConA-mediated liver damage can be prevented by blocking antibodies against IFN-γ rather than TNF, suggesting that IFN-γ is an essential key regulator in ConA-induced liver injury (Tagawa et al., 1997). The transcription factor STAT1, which is associated with the IFN-*γ* signaling pathway, is crucial for ConA-induced hepatitis. Activation of STAT1 by IFN-γ is significant for the induction of T-bet during Th1 cells differentiation (Siebler et al., 2003; Jaruga et al., 2004). Therefore, we investigated the effect of Sophocarpine on the activation of STAT1 and the expression of T-bet in ConA-induced hepatitis. In the present study, Sophocarpine effectively inhibited STAT1 synthesis and activation as well as the expression of T-bet (**Figure 5**). SOCS1 has been shown to suppress inflammation and is indispensable for the negative regulation of STAT1 (Hashimoto et al., 2009). Therefore, gene expression analysis of the SOCS1 protein in the mouse livers was performed. The results indicated that Sophocarpine downregulated the expression of T-bet [Control: 27.44 ± 11.55, Model: 67.74 ± 10.58, Sophocarpine (30 mg⋅kg^-1^): 36.81 ± 4.51, Sophocarpine (60 mg⋅kg^-1^): 27.99 ± 5.03] via inhibition of STAT1 activation [Control: 24.22 ± 14.78, Model: 205.49 ± 21.08, Sophocarpine (30 mg⋅kg^-1^): 75.72 ± 16.64, Sophocarpine (60 mg⋅kg^-1^): 8.21 ± 3.34] and overexpression of SOCS1 [Control: 1.00 ± 0.58, Model: 1.76 ± 0.31, Sophocarpine (30 mg⋅kg^-1^): 2.21 ± 0.12, Sophocarpine (60 mg⋅kg^-1^): 3.83 ± 0.69], suggesting that Sophocarpine decreased the release of IFN-γ by inhibiting the activation of the IFN-γ/STAT1 signaling pathway and Th1 differentiation.

## Discussion

An increasing number of traditional Chinese herbal medicines have been reported to serve as potential therapeutic protection against liver disease (Ding et al., 2012; Hu et al., 2013). Sophocarpine is a quinolizidine alkaloid extracted from *Radix Sophorae Subprostratae* a traditional Chinese herb that has been widely used in the treatment of liver diseases for years in China. In previous studies, Sophocarpine has been reported to significantly decrease the production of inflammatory cytokines and attenuate liver injury induced by Poly I: C/D-GalN and suppressed NK cell activation (Huang et al., 2016).

In the present study, we showed that Sophocarpine could protect against T cell-dependent immune hepatitis induced by ConA. Pretreatment with Sophocarpine significantly and dose-dependently downregulated the levels of ALT, AST, and TBIL. Histological staining significantly demonstrated that liver inflammation and apoptotic necrosis was dramatically reduced in Sophocarpine-pretreated mice. Sophocarpine also significantly reduced the release of chemokines and adhesion molecules as well as pro-inflammatory cytokines.

Con A is a well-known T-cell mitogen, after ConA administration, the levels of inflammatory cytokine is elevate subsequently resulting in abundant liver necrosis with mass lymphocytes infiltration (Kato et al., 2013). Chemokines and adhesion molecules play important roles in inflammatory and immune responses in ConA-induced hepatitis (Luo et al., 2015). Chemotactic factors was activated which attracted leukocytes into the liver to amplify the inflammatory response (Xu et al., 2008). MIP-1α has been shown to significantly contribute to the pathogenesis of ConA-induced hepatitis as hepatic injury was strikingly attenuated in mice with MIP-1α deficiency (Ajuebor et al., 2004). In addition, CXCL10 has been reported to be a specific chemoattractant for T lymphocytes, and the expression of CXCL10 was specifically induced in the livers of mice treated with ConA (Tamaru et al., 2000). Furthermore, ICAM-1 has been reported to contribute to the development of hepatitis by mediating adhesion and supporting the migration of lymphocytes into the liver (Kawasuji et al., 2006). In the present study, we found that Sophocarpine inhibited MIP-1α, CXCL10, and ICAM-1 mRNA expression, which in turn inhibited the infiltration of leukocytes, thereby alleviating the liver damage induced by ConA.

IFN-γ has been reported to be a critical mediator of liver injury in the pathogenesis of ConA-induced hepatitis (Kusters et al., 1996). ConA-mediated liver damage may be prevented by blocking IFN-γ (Tagawa et al., 1997). Furthermore, IFN-γ is a major cytokine responsible for STAT1 activation. Disruption of the IFN-γ or STAT1 genes abolished elevated ALT activities and necrosis, suggesting that IFN-γ/STAT1 plays an essential role in ConA-induced hepatitis (Hong et al., 2002). In this signaling pathway, SOCS1 was considered as an important negative regulation of the cytokine-JAK–STAT pathway and forced expression of SOCS1 could prevented ConA-induced liver injury by suppressing STAT1 activation (Torisu et al., 2008; Yoshimura, 2009). Constitutive activation of STAT1 as well as constitutive expression of IFN-γ-inducible genes was observed in SOCS1 KO mice. Furthermore, T cell-specific SOCS1deficient mice demonstrated an enhanced sensitivity to ConA-induced hepatitis (Hashimoto et al., 2011; Yoshimura et al., 2012). In the current study, Sophocarpine pretreatment significantly inhibited STAT1 activity and increased the expression of SOCS1, subsequently downregulating its signaling proteins T-bet and inhibiting the production of Th1 cytokines, which protects mice from ConA-induced acute hepatitis. However, the precise mechanism underlying this process remains unknown.

## Conclusion

In summary, we used the T cell-dependent hepatitis mouse model to examine the protective effects of Sophocarpine. In the present study, Sophocarpine attenuated ConA-induced liver injury in mice. The protective effect of Sophocarpine was associated with its inhibition of inflammatory mediators, chemokines, and the STAT1 signaling pathway. Taken together, the results of this study provide new opportunities for the use of Sophocarpine in the prevention and treatment of immune-mediated liver disease.

## Author Contributions

Z-FB, J-BW, and X-HX contributed to research design; X-XS performed the research; S-JL, H-HS, and R-LW provided innovative views and opinions; Y-MZ, Y-MG, and MN analyzed the data; and X-XS and C-EZ wrote the manuscript.

## Conflict of Interest Statement

The authors declare that the research was conducted in the absence of any commercial or financial relationships that could be construed as a potential conflict of interest.

## Abbreviations

ALT: alanine aminotransferase
AST: aspartate aminotransferase
ConA: Concanavalin A
CXCL10: chemokine ligand 10
ICAM-1: intercellular adhesion molecule-1
IFN-γ: interferon-γ
IL: interleukin
LPS: lipopolysaccharide
MIP-1α: macrophage inflammatory protein-1α
SOCS1: suppressor of cytokine signaling 1
STAT1: signal transducers and activators of transcription1
TBIL: total bilirubin
TGF-β1: transforming growth factor-β1
TNF-α: tumor necrosis factor-α
